# Severe bilateral pleuropneumonia caused by *Legionella sainthelensi*: a case report

**DOI:** 10.1186/s12879-021-06651-1

**Published:** 2021-09-17

**Authors:** Laure Kamus, Bénédicte Roquebert, Jérôme Allyn, Nicolas Allou, Dorothée Valance, Charles Simon, Marie-Christine Jaffar-Bandjee, Ghislaine Descours, Sophie Jarraud, Guillaume Miltgen

**Affiliations:** 1 Laboratoire de Bactériologie, CHU Félix Guyon, Allée des Topazes, 97400 Saint-Denis, La Réunion, France; 2 UMR Processus Infectieux en Milieu Insulaire Tropical, CNRS 9192, INSERM U1187, IRD 249, Université de La Réunion, Saint-Denis, La Réunion, France; 3 Service de Réanimation Polyvalente, CHU Félix Guyon, Saint-Denis, La Réunion, France; 4 Département d’Informatique Clinique, CHU Félix Guyon, Saint-Denis, La Réunion, France; 5 Service de Pneumologie, CHU Félix Guyon, Saint-Denis, La Réunion, France; 6 Centre National de Référence des Légionelles, Institut des Agents Infectieux, Hospices Civils de Lyon, Lyon, France; 7 CIRI, Centre International de Recherche en Infectiologie, Legionella pathogenesis Team, Univ Lyon, Inserm U1111, Université Claude Bernard Lyon 1, CNRS, UMR5308, ENS de Lyon, Lyon, France

**Keywords:** bilateral pleuropneumonia, *Legionella sainthelensi*, Legionnaires’ disease, PCR syndromic testing, Case report

## Abstract

**Background:**

*Legionella spp.* are ubiquitous freshwater bacteria responsible for rare but potentially severe cases of Legionnaires’ disease (LD). *Legionella sainthelensi* is a non-*pneumophila Legionella* species that was first isolated in 1980 from water near Mt. St-Helens (USA). Although rare cases of LD caused by *L. sainthelensi* have been reported, very little data is available on this pathogen.

**Case presentation:**

We describe the first documented case of severe bilateral pleuropneumonia caused by *L. sainthelensi*. The patient was a 35-year-old woman with Sharp’s syndrome treated with long-term hydroxychloroquine and corticosteroids who was hospitalized for an infectious illness in a university hospital in Reunion Island (France). The patient’s clinical presentation was complicated at first (bilateral pneumonia, multiloculated pleural effusion, then bronchopleural fistula) but her clinical condition eventually improved with the reintroduction of macrolides (spiramycin) in intensive care unit. Etiological diagnosis was confirmed by PCR syndromic assay and culture on bronchoalveolar lavage.

**Conclusions:**

To date, only 14 documented cases of *L. sainthelensi* infection have been described worldwide. This pathogen is difficult to identify because it is not or poorly detected by urinary antigen and molecular methods (like PCR syndromic assays that primarily target *L. pneumophila* and that have only recently been deployed in microbiology laboratories). Pneumonia caused by *L. sainthelensi* is likely underdiagnosed as a result. Clinicians should consider the possibility of non-*pneumophila Legionella* infection in patients with a compatible clinical presentation when microbiological diagnostic tools targeted *L. pneumophila* tested negative.

## Background

*Legionella spp.* are ubiquitous Gram-negative freshwater bacteria that can cause sporadic or epidemic human infections, notably severe pneumonia. The main species responsible for Legionnaires’ disease (LD) is *Legionella pneumophila*, which accounts for more than 95% of community-acquired LD worldwide [1, 2]. The most frequently reported non-*pneumophila Legionella* (N*P*-*L*) species are *L. longbeachae*, *L. micdadei*, *L. bozemanii*, and *L. dumoffii.* Less common is *Legionella sainthelensi*, which was first isolated in 1980 from water near Mt. St-Helens (USA) [1, 3]. Here we report a case of *L. sainthelensi* infection in Reunion Island, a French overseas department and subtropical volcanic island located in the Southwest Indian Ocean, close to Madagascar. This is the first documented case of severe bilateral pleuropneumonia caused by *L. sainthelensi*.

## Case presentation

A 35-year-old woman was admitted to Reunion Island University Hospital for an infectious syndrome with febrile dyspnea. Her medical history was mainly a Sharp’s syndrome treated with long-term hydroxychloroquine and corticosteroids. She had not travelled abroad in the past 2 years. On admission, the patient presented with Systemic Inflammatory Response Syndrome (fever 39.7 °C, pulse 120 beats/min, blood pressure 112/66 mmHg, respiratory rate 32 breaths/min, and oxygen saturation in room air 100%). Pulmonary clinical signs were pleuritic pain, dyspnea, dry cough, and crackles. The patient had no extrapulmonary symptoms. Laboratory investigations showed biological inflammatory reaction, with a white blood cell count of 8.6 G/L (neutrophils: 7.65 G/L), lymphopenia (0.54 G/L), anemia (10.2 g/dL), and elevated C-reactive protein (76 mg/dL). On Day 1, an injected chest computed tomography (CT) scan was performed showing left basal pleuropneumonia with pleural effusion, subpleural nodular lesions, and lymphadenopathies. Three doses of spiramycin (3 M IU) were administered and antimicrobial therapy with ceftriaxone (3 g per day) was initiated. The patient was transferred to the Internal Medicine department. On Day 3, her respiratory status and biological inflammatory reaction worsened, with hyperleukocytosis of 15.2 G/L (neutrophils: 14.2 G/L) and elevated C-reactive protein (> 350 mg/dL); however, she presented no signs of septic shock. Urinary antigen tests for *L. pneumophilia* and *Streptococcus pneumoniae* were negative. Antimicrobial therapy with spiramycin (3 M IU every 8 h) was reintroduced. The patient was transferred to intensive care unit (ICU). Treatment was changed to triple antimicrobial therapy with piperacillin-tazobactam (16 g per day), spiramycin (3 M IU every 8 h), and amikacin (30 mg per kg). Complementary microbiological investigations performed in ICU on blood, urine, cerebrospinal fluid, and sputum were all negative. Respiratory multiplex PCR assay on nasopharyngeal swab (FTD Respiratory pathogens-21, Fast Track Diagnostics, Luxembourg; not targeting atypical microorganisms) was negative. A control injected chest CT-scan (Day 5) revealed multiple nodular parenchymal lesions in both lobes, as well as increasing multiloculated pleural effusion. On Day 6, the patient’s clinical condition finally improved. She was transferred to the Pneumology department for etiological investigations.

**Fig. 1:**
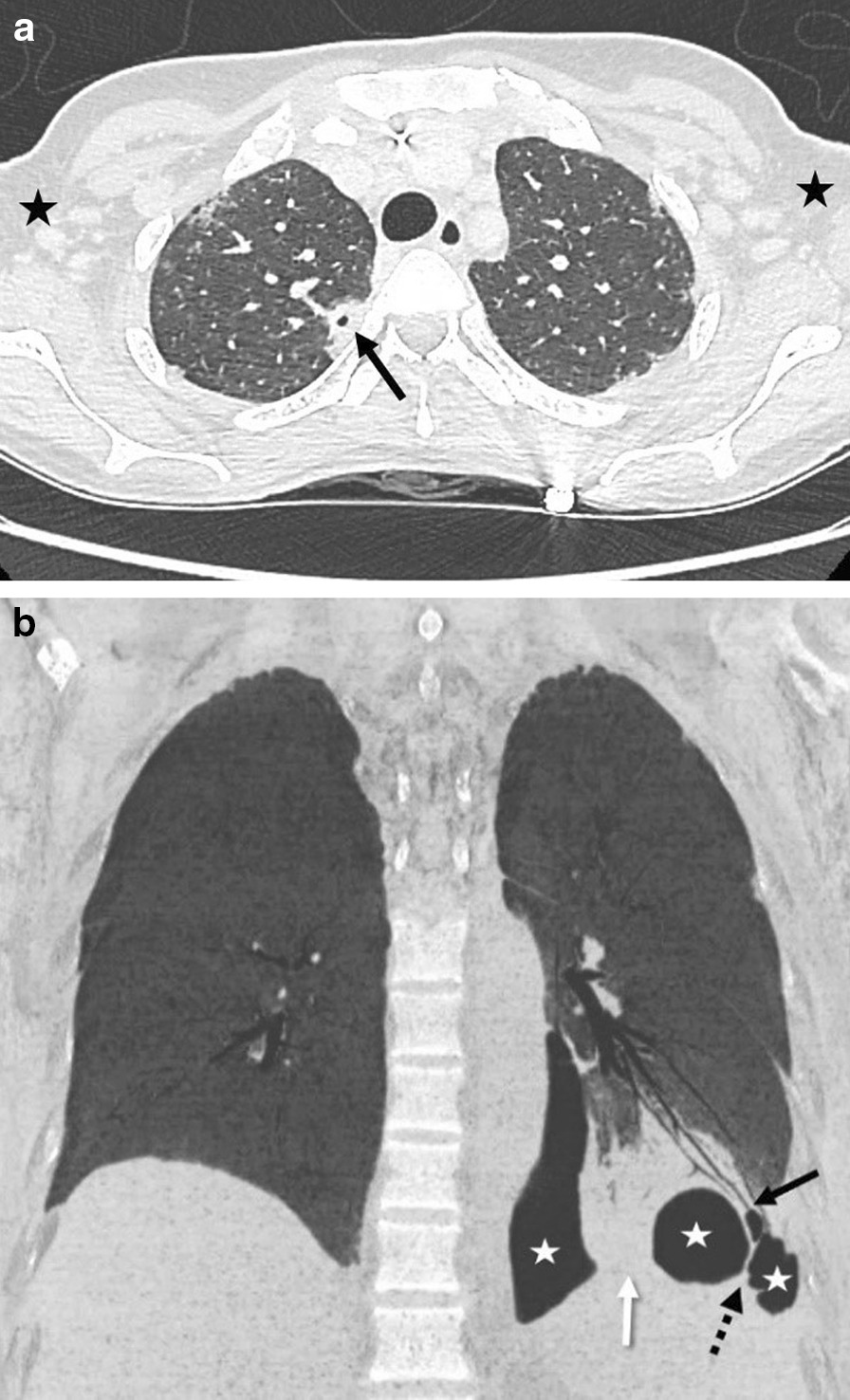
Injected chest CT scan showing bilateral pleuropneumonia with left bronchopleural fistula (second control CT scan performed on Day 10). **a** Axial slice of injected thoracic CT scan in parenchymal window. Right upper lobe parenchymal nodular lesion with central necrosis (black arrow). Bilateral axillary lymphadenomegalies (black stars). **b** Frontal minimal intensity projection image of thoracic CT scan in parenchymal window showing left lower lobe pneumonia (white arrow) with bronchopleural fistula (black arrow) and multiloculated pneumothorax (white stars) with septa (dotted arrow)

On Day 9, a specific PCR assay (FTD Atypical CAP, Fast Track Diagnostics, Luxembourg and Genesig Advanced Kits, Primerdesign Ltd, UK) on bronchoalveolar lavage (BAL) was positive for *Legionella spp.* (cycle threshold-Ct-34.35) and negative for *L. pneumophila.* The BAL culture was positive with typical colonies of *Legionella spp.* which were identified as *L. sainthelensi* or *L. santicrucis* using MALDI-TOF Biotyper (Bruker Daltonics, Bremen, Germany). A second control CT-scan performed on Day 10 showed the persistence of multiloculated pleural effusion and the appearance of bronchopleural fistula in the left lower lobe (Fig. 1a and b). Pleural fluid collected on Day 5 was retrospectively reanalyzed and tested positive for *Legionella spp*. (Ct 28.40). Biochemical analyses of pleural fluid showed parapneumonic pleural effusion with excudate (total protein: 49 g/L; LDH: 1156 IU/L; pH: 7.3 and glucose 2.1 mmol/L).

The French National Reference Center for *Legionella* confirmed the identification of *L. sainthelensi* by MIP sequencing [4]. Raw reads from whole-genome sequencing using Nextera XT technology (Illumina, https://www.illumina.com) were deposited into the European Nucleotide Archive (study Accession No. PRJEB40106).

Minimum inhibitory concentrations (MICs) determined using the broth microdilution method were similar to those reported for wild-type *L. pneumophila* strains (0.032 mg/L for moxifloxacin, 0.016 mg/L for levofloxacin, 0.063 mg/L for erythromycin and azithromycin, and 0.001 mg/L for rifampicin) [5].

Three weeks after admission, the patient’s general health improved and she was sent home.

## Discussion and conclusions

Here, we reported the first case of severe bilateral pleuropneumonia caused by *L. sainthelensi*. To our knowledge, only 14 documented cases of *L. sainthelensi* infection have been reported to date, 9 of which were detected during 2 outbreaks in Canada in 1994 (Table 1) [6–9]. Interestingly, as in the case reported here, several environmental strains of *L. sainthelensi* have been found in volcanic environments [10].

**Table 1:** Literature review of documented cases of human infection with *L. sainthelensi* since the pathogen was first described 1980

References	Year	Number of case(s)	Country of diagnosis	Age	Sex	Comorbidity	Type of sample	Technique of identification	Antibiotic therapy	Death
[6]	1989	1	Georgia, USA		Male		Pleural fluid	Culture on specific agar (BCYE^1^)		
[6]	1989	1	Virginia, USA		Male		Sputum	Culture on specific agar (BCYE^1^)		
[6]	1989	1	California, USA	50	Male	Diabetes, alcohol, tobacco, renal failure, chronic lung disease	Bronchoalveolar lavage (BAL) fluid	Culture on specific agar (BCYE^1^)	Erythromycin	Yes
[8]	1994	9	Ontario, Canada	69–102			Serum	Serologic testing for *L. sainthelensi*-specific antibody^2^		
[7]	2002–2014	1	Texas, USA	28	Male	Acute leukemia, hematopoietic stem cell transplant, graft-versus-host disease	BAL fluid	Direct fluorescent antibody assay for *Legionella* species	Ceftriaxone, azithromycin, gatifloxacin	No
[9]	2001	1	Christchurch, New Zealand					Culture on specific agar (BCYE)		

^a^ BCYE, Buffered Charcoal Yeast Extract

^b^ Fourfold-rise in *L. sainthelensi* serogroup 1 serum antibodies to a titer ≥ 1/128

New molecular biology tools, and in particular PCR syndromic assays targeting atypical microorganisms, can help confirm the diagnosis of pneumonia caused by N*P*-*L* species. Indeed, some marketed kits are able to detect these species even though they are not specifically designed to do so (*i.e.* Genesig or FTD targeting *L. pneumophila* and *L. longbeachae*). However, they are rarely used at the moment because their introduction is fairly recent and because they are costly and expertise-demanding. In this context, the number of cases of pneumonia caused by N*P*-*L* species is likely underestimated.

This case report indicates that good communication between medical practitioners and clinical microbiologists is needed to ensure the best complementary investigations are performed in cases of clinical suspicion of LD. As regards our patient, a search for *Legionella spp.* would likely have allowed an earlier etiological diagnosis. Although culture has limited sensitivity, strain isolation is required for molecular diagnosis confirmation and for epidemiological monitoring by national and international networks [11, 12].

Given the limited availability of data, it is difficult to determine whether infections caused by *L. sainthelensi* are more severe than those caused by other *Legionella* species (notably *L. pneumophila*). It should be noted, however, that a case-fatality rate of 13.8% was reported in the only 2 documented outbreaks linked to *L. sainthelensi* (in Canada). This is slightly higher than the case-fatality rate of 8–9% reported by various surveillance networks [8, 11, 12]. Lastly, our case report reveals a new category of at-risk patients: namely, young people receiving immunosuppressive treatment [13].

Our patient tested negative twice on the urinary antigen test. This is likely because the test specifically targeted *L. pneumophila* serogroup 1, which accounts for the great majority of LD cases worldwide (95.4% in France) [11]. Accordingly, in cases of strong clinical or radiological suspicion of LD, and especially in the presence of severity criteria, treatment with antibiotics targeting atypical germs (*i.e.* *Legionella spp.*, *Chlamydophila pneumoniae*, and *Mycoplasma pneumoniae*) should be continued even when the urinary antigen test is negative [14]. In the case of our patient, the right decision was made in reintroducing macrolides when her clinical condition began to deteriorate. Although spiramycin is not recommend as first-line therapy, its use is accepted by French and European guidelines because it causes less drug interactions than the recommended treatment (azithromycin and levofloxacin) and because it is available in injectable form in France [15].

Clinicians should be aware that LD can be caused by N*P*-*L* species, which are not detected by urinary antigen testing. At the moment, pneumonia caused by *L. sainthelensi* is likely underdiagnosed, as the few PCR syndromic assays that can detect N*P*-*L* species are rarely used at the moment. Dialogue between clinicians and microbiologists and PCR assays targeting all *Legionella spp.* are needed to detect N*P*-*L*, which can be responsible for severe (albeit rare) cases of LD.

## Data Availability

New genome sequence obtained in this study was deposited into the European Nucleotide Archive under Accession Numbers PRJEB40106 (https://www.ebi.ac.uk/ena/browser/view/PRJEB40106).

## Abbreviations

BAL: Bronchoalveolar lavage
CT: Computed tomography
Ct: Cycle threshold
ICU: Intensive care unit
IU: International unit
LD: Legionnaires’ disease
MIC: Minimum inhibitory concentration
N*P*-*L*: Non-*Pneumophila Legionella* species
PCR: Polymerase chain reaction
